# Acute glycemic responses to meal-associated alcoholic beverages and ethanol in adults with type 2 diabetes: a systematic review

**DOI:** 10.3389/fendo.2026.1967515

**Published:** 2026-09-29

**Authors:** Jiahao Li, Huizhi Yang

**Affiliations:** School of Sports Medicine and Rehabilitation, Beijing Sport University, Beijing, China

**Keywords:** alcohol, ethanol, fasting glucose, nocturnal hypoglycemia, postprandial glucose, systematic review, type 2 diabetes, wine

## Abstract

**Background:**

A lower next-morning glucose value after evening alcohol consumption may be mistaken for improved diabetes control, but direct evidence for this observation and for preceding nocturnal hypoglycemia is limited. We evaluated acute postprandial, overnight, and next-morning glycemic responses to alcoholic beverages or ethanol in adults with type 2 diabetes.

**Methods:**

PubMed, Web of Science Core Collection, the Cochrane Library, MEDLINE with Full Text, SPORTDiscus with Full Text, eBook Collection, OpenDissertations, and Embase were searched from inception to 28 August 2026. Acute alcohol-free comparisons and active comparisons between alcohol-containing beverages were eligible, with the latter classified only as beverage-composition evidence. Risk of bias was assessed with RoB 2 or ROBINS-I, and findings were synthesized narratively.

**Results:**

Fifteen small, heterogeneous studies were included. Meal-associated exposures did not consistently reduce postprandial glucose. Two active-beverage studies reinforced the importance of beverage composition: sweet versus dry wine and sherry produced no significant difference in evening glucose AUC, while glucose increased after carbohydrate-containing beer and sake but remained near baseline after carbohydrate-free shochu in a fasting comparison. Neither study estimated alcohol versus no alcohol. Only one alcohol-versus-control crossover study (n = 16) reported lower next-morning glucose (paired difference, −0.7 ± 0.2 mmol/L); the value was not hypoglycemic and the finding was not independently replicated. No study documented that a lower morning value was preceded by nocturnal hypoglycemia, and none reported contemporary overnight continuous-glucose-monitoring metrics sufficient to exclude brief or asymptomatic events.

**Conclusion:**

Very-low-certainty evidence does not support alcoholic beverages or ethanol as a strategy to improve acute glycemia or lower morning glucose. Whether evening consumption contributes to delayed nocturnal hypoglycemia remains unresolved, especially in insulin- or insulin-secretagogue-treated populations.

**Systematic review registration:**

https://www.crd.york.ac.uk/prospero/view/, identifier CRD420261474632.

## 1 Introduction

Postprandial and fasting glucose values are highly visible indicators of day-to-day diabetes management. Anecdotal reports and clinical observations suggest that fasting glucose may appear unexpectedly lower on the morning after alcohol is consumed with an evening meal. This apparently favorable value can be misinterpreted as evidence that alcohol improves glycemic control. However, a transient reduction in glucose is not equivalent to improved glucose regulation, and a lower next-morning value cannot by itself distinguish metabolic benefit from an overnight glucose disturbance.

This ambiguity is biologically plausible because alcohol can influence glucose homeostasis in opposing directions. Ethanol metabolism suppresses hepatic gluconeogenesis, alters substrate availability, and may modify insulin sensitivity and secretion (1). During the overnight fasting period, these effects may influence glucose availability and counterregulatory responses. The clinical relevance may be greater in people using insulin or insulin secretagogues, during prolonged fasting, or after physical activity (2, 3). Conversely, alcoholic beverages may add carbohydrate and energy, while beverage composition and the accompanying meal may attenuate or reverse an ethanol-related glucose-lowering effect. The same next-morning glucose value could therefore arise from substantially different metabolic pathways.

The experimental literature has not been organized around this interpretation problem. Studies have used wine, spirits, or isolated ethanol; alcohol doses range from moderate fixed amounts to 1 g/kg; and outcomes include postprandial glucose curves, incremental area under the curve (iAUC), next-morning fasting glucose, continuous glucose monitoring (CGM), hypoglycemic clamps, and mechanistic indices. Some experiments combine alcohol with exercise or investigational drugs. As a result, evidence relevant to the clinical meaning of lower morning glucose is distributed across studies of different directness and cannot be understood through statistical significance alone.

The primary question of this systematic review was: in adults with type 2 diabetes, how does alcohol consumed with meals affect postprandial glucose responses and overnight glucose stability? The secondary question was: when lower next-morning glucose is observed after evening alcohol consumption, does the available evidence indicate improved glucose regulation, a transient metabolic effect, or preceding nocturnal hypoglycemia? This review was therefore not designed to determine whether alcohol can be used to lower glucose, but to clarify the clinical meaning of apparently favorable glucose values after meal-associated alcohol consumption.

The physiological interpretation requires particular caution. A falling nocturnal glucose concentration would ordinarily activate glucagon and catecholamine counterregulation and may be followed by compensatory glucose recovery or elevation. Alcohol may simultaneously suppress hepatic gluconeogenesis and attenuate the glucagon response (1, 2). Thus, if an overnight decline occurred, the next-morning value might remain relatively low rather than showing a clear compensatory rise. This pathway is biologically plausible but remains hypothetical in this population: the included studies did not directly demonstrate the sequence of nocturnal hypoglycemia, impaired counterregulation, and persistently lower morning glucose.

## 2 Methods

### 2.1 Reporting framework and protocol

This systematic review was conducted and reported in accordance with PRISMA 2020 (4), and the narrative synthesis followed SWiM guidance (5). The review was registered in PROSPERO on 10 August 2026 (CRD420261474632; https://www.crd.york.ac.uk/prospero/view/CRD420261474632). Database searches covered the period from inception to 28 August 2026. Acute comparisons between two alcohol-containing beverages were classified as beverage-composition evidence and were not interpreted as estimates of alcohol versus abstinence.

### 2.2 Information sources and search strategy

The searches identified 489 records from PubMed, 874 from Web of Science Core Collection, 402 from the Cochrane Library, 159 from four EBSCOhost databases (MEDLINE with Full Text, SPORTDiscus with Full Text, eBook Collection [EBSCOhost], and OpenDissertations), 3,257 Embase article records, and 126 Embase Clinical Trials register records. All databases were searched from inception to 28 August 2026. Search concepts covered type 2 diabetes, ethanol and alcoholic beverages, and glucose or glycemic outcomes. Controlled vocabulary and database-specific free-text syntax were used, with no publication-year restriction. Reference lists of eligible reports and relevant reviews were checked. The complete reproducible strategies are provided in **Supplementary Table S1**.

For four result-bearing reports that remained unavailable, title, author, bibliographic, identifier, and publisher-link searches were repeated in the searched resources and publicly accessible sources. Corresponding authors were contacted by email, but no response had been received by 31 August 2026. Two additional trial-register records had no posted results or linked result-bearing publication and were not treated as studies. All unavailable and registry-only records are identified in **Supplementary Table S6**.

### 2.3 Eligibility criteria

The review question framework is summarized in **Table 1**. Studies were eligible when they (1): enrolled adults with type 2 diabetes or reported separately extractable type 2 diabetes data (2); evaluated a single oral alcohol challenge with clinical or mechanistic outcomes assessed within 24 h; and (3) reported at least one postprandial, nocturnal, next-morning, hypoglycemic, or directly relevant mechanistic glycemic outcome. The primary comparison was an alcohol-containing exposure versus a concurrent or within-participant alcohol-free condition. Acute within-participant comparisons between two alcohol-containing beverages were also eligible when they informed beverage carbohydrate or matrix effects; these studies were explicitly separated from alcohol-versus-no-alcohol evidence. Randomized or non-randomized crossover, repeated-measures, parallel, quasi-experimental, clamp, and pharmacodynamic studies were eligible. Mixed diagnostic samples were excluded when type 2 diabetes results could not be separated.

**Table 1:** PICO framework and review question.

Element	Definition
Population	Adults (≥18 years) with type 2 diabetes mellitus; mixed diagnostic samples were eligible only when type 2 diabetes data were separately extractable.
Intervention/exposure	Acute oral alcohol or alcoholic-beverage exposure. Meal-associated studies constituted the direct clinical evidence; fasting, exercise, drug-interaction, FSIGT, and clamp studies were retained only as indirect mechanistic or special-context evidence.
Comparator	Primary: alcohol-containing exposure versus an alcohol-free concurrent or within-participant condition. Active comparisons between alcohol-containing beverages were eligible only for beverage-composition evidence after the 28 August 2026 amendment.
Outcomes	Postprandial glucose; overnight glucose stability/hypoglycemia; next-morning fasting glucose; and relevant mechanistic outcomes.
Study designs	Randomized or non-randomized crossover, repeated-measures, parallel, quasi-experimental, clamp, and pharmacodynamic studies.

We excluded reviews, animal or *in vitro* experiments, chronic drinking studies, mixed populations without separately extractable type 2 diabetes data, and reports without relevant glycemic outcomes. Direct clinical evidence comprised meal-associated studies measuring postprandial, overnight, or next-morning glucose. Active-beverage comparisons without an alcohol-free condition were used only to examine beverage composition. Exercise, fasting beverage, and drug-interaction studies were classified as special-context indirect evidence, whereas fasting intravenous glucose tolerance tests and hypoglycemic clamps were classified as mechanistic indirect evidence. Indirect and active-comparator studies were not assigned evidentiary weight equivalent to direct alcohol-versus-no-alcohol meal studies.

### 2.4 Study selection

Records were deduplicated before title, abstract, and full-text screening. Two reviewers (J.L. and H.Y.) independently assessed records at each stage. The screening log recorded a single primary reason for exclusion at each stage. Disagreements were resolved through discussion and consensus.

### 2.5 Data extraction and outcome hierarchy

A standardized form was used to extract study design, country, sample size, age, sex, body mass index, diabetes duration, baseline HbA1c and glucose, glucose-lowering treatment, beverage type and volume, ethanol dose, beverage carbohydrate content, comparator, standardized meal composition and timing, fasting status, observation window, glucose measurement method, study-specific hypoglycemia definition or ascertainment, clinical glycemic outcomes, mechanistic outcomes, and adverse events. Items not reported were recorded as not reported rather than inferred. Values were retained in their original statistical form and converted to common glucose units where appropriate. Glucose values in mg/dL were divided by 18 to obtain mmol/L; the concentration component of glucose AUC values was converted similarly while retaining the original time dimension.

Two reviewers (J.L. and H.Y.) independently extracted the data and assessed risk of bias. Discrepancies were resolved by checking the full report and reaching consensus.

For postprandial synthesis, AUC or iAUC was prioritized, followed by 2-h glucose and then the author-defined peak excursion. For nocturnal outcomes, minimum glucose, time below range, and hypoglycemic events were prioritized; study-specific thresholds and ascertainment methods were retained because the older reports did not consistently use the contemporary <3.9 mmol/L threshold. For next-morning outcomes, fasting glucose and the paired condition difference were prioritized. One prioritized result per study was used within each synthesis domain; multiple time points from the same participants were described without being treated as independent observations.

### 2.6 Risk-of-bias assessment

Randomized trials were assessed using Cochrane RoB 2; the crossover version was used for randomized crossover studies and included consideration of period and carryover effects (6). Non-randomized intervention studies were assessed using ROBINS-I (7). Assessments targeted the glycemic result most relevant to this review rather than assigning a single generic score to each publication. No numerical quality score was calculated.

### 2.7 Data synthesis

Before deciding against meta-analysis, we examined whether direct meal-associated studies formed clinically comparable subsets. No subset of at least two studies used sufficiently similar beverage or ethanol exposure, comparator, meal composition, outcome definition, assessment time, and reporting format while also providing a paired-condition variance or statistics from which it could be derived. Separate-condition SDs or SEs were not pooled as though the crossover studies were parallel trials because doing so would discard within-participant correlation. Corresponding authors of the four unavailable reports were contacted to request full texts, but authors of the included studies were not contacted specifically to obtain missing paired-difference variances or within-participant correlations. Sensitivity analyses using assumed correlations were not performed because the very small, clinically heterogeneous subsets would have produced assumption-driven estimates.

Narrative synthesis followed a prespecified outcome hierarchy and separated direct clinical, special-context, and mechanistic evidence. Effect direction was classified relative to the alcohol-free comparator as lower, no clear difference, or higher; magnitude was retained in the reported or harmonized units. For active-beverage studies without an alcohol-free condition, effect direction was classified only between the alcohol-containing beverage conditions and was synthesized separately as beverage-composition evidence. Conclusions considered effect magnitude, sample size, precision, consistency, directness, and risk of bias rather than statistical significance or a count of positive studies. Structured effect-direction and heterogeneity information is provided in **Supplementary Table S7**. Certainty was assessed with GRADE considerations and is reported in **Supplementary Table S5**.

## 3 Results

### 3.1 Study selection

The searches identified 5,181 bibliographic records (PubMed, n = 489; Web of Science Core Collection, n = 874; Cochrane Library, n = 402; EBSCOhost, n = 159; Embase Articles, n = 3,257) and 126 Embase Clinical Trials register records. After removal of 1,104 duplicate database records, 4,077 records underwent title screening; 299 reviews or non-original reports and 97 animal or *in vitro* records were excluded. Of 3,681 abstracts assessed, 3,660 were excluded for population (n = 2,000), intervention/content (n = 809), study design (n = 804), or outcome (n = 47). Twenty-one database reports were sought, four were not retrieved, and 17 were assessed; one mixed type 2 diabetes/prediabetes report with inseparable type 2 diabetes data (9) and one registry/protocol-only record without results were excluded. Of 126 trial-register records, two overlapped with database records, 123 were excluded as irrelevant, and one additional record had no posted results or linked publication. Fifteen studies were included (**Figure 1**). **Supplementary Table S6** identifies unavailable and excluded reports.

**Figure 1:**
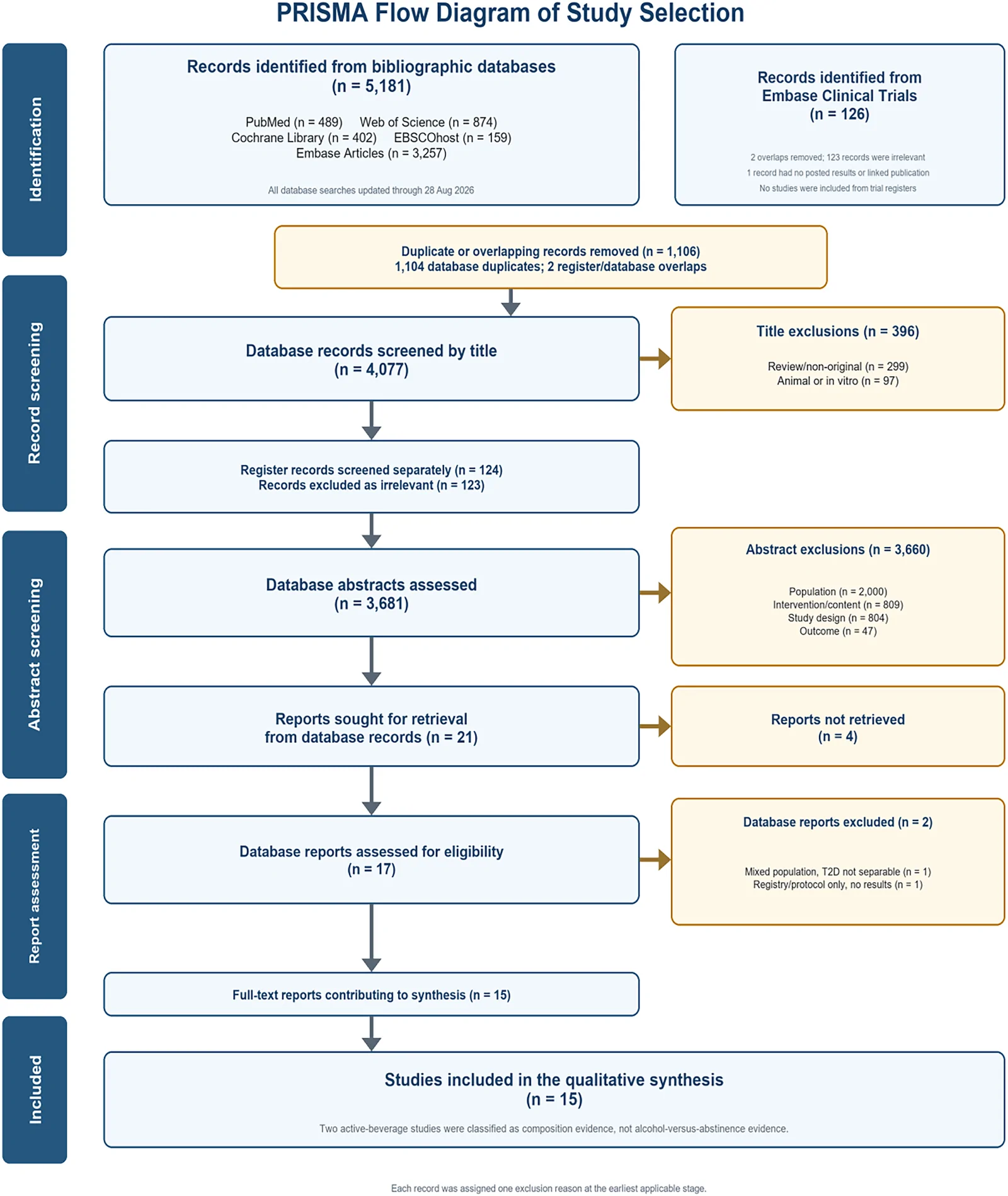
PRISMA flow diagram of study selection.

### 3.2 Study characteristics

The 15 included studies were published between 1990 and 2016; eight were published before 2000, and the newest included study was published in 2016. Eight studies provided direct meal-associated clinical evidence, four were special-context studies involving exercise, fasting beverage comparisons, or drug interactions, and three were mechanistic FSIGT or clamp studies. Samples were small (6–30 participants), generally metabolically stable, and predominantly managed with diet or oral agents; insulin-treated and insulin-secretagogue-treated populations were poorly represented. Detailed participant characteristics are shown in **Table 2** and **Supplementary Table S2**.

**Table 2:** Characteristics of included studies.

Study/country	Participants/design/treatment	Exposure and comparator	Meal/timing/window	Outcomes and hypoglycemia ascertainment	Evidence role
Rasmussen 1999 (8) Denmark	n=12 meal phase/n=11 fasting phase; middle-aged; 7 men/4 women in fasting phase; BMI ~29; diet/oral treatment; randomized crossover	0.4 g/kg ethanol ± exercise; rest/exercise controls	Light meal (48% carbohydrate, 38% fat, 14% protein); 4 h	Glucose iAUC/nadir; acute events only; no overnight threshold	Special context: exercise
Bantle 2008 (10) USA	n=18; non-insulin-treated T2D; random-order crossover; other characteristics NR in main report summary	Wine 240 mL (24 g ethanol) vs grape juice	Dinner to next morning	Glucose/insulin curves; no nocturnal nadir or time-below-range definition	Direct overnight
Dalgaard 2004 (11) Denmark	n=11 (5 women/6 men); diet/oral agents; insulin excluded; three-meal crossover	40 g ethanol with 50 g carbohydrate vs isocaloric 120 g carbohydrate meal	High-fat mixed meal; 8 h	Glucose and incretins; hypoglycemia not prespecified	Direct meal; composition mismatch
Gin 1999 (12) France	n=30; age 53 ± 6 y; BMI 27 ± 2; diet/oral agents; three subgroups with self-control	Red wine 200 mL, ethanol 16 g, or tannic acid; water/control meal	Lunch: 600 kcal, 65 g carbohydrate, 20 g fat, 34 g protein; 5 h	Peak excursion/time points; hypoglycemia not defined	Direct postprandial
Christiansen 1994 (13) Denmark	n=10; randomized crossover; participant details NR here	24 g ethanol vs control beer	Standardized light meal; 4 h	Glucose iAUC; hypoglycemia not defined	Direct postprandial
Schaller 2010 (14) Austria	n=12 men; age 64 ± 6 y; BMI 28.4 ± 5.7; HbA1c ~7.4%; diet/oral agents; open self-control	Vodka providing 40 g ethanol vs baseline/control period	Fasting FSIGT	Insulin sensitivity and glucose effectiveness; not a clinical hypoglycemia study	Mechanistic
Zhi 2016 (15) Multicenter	n=18; T2D; BMI eligibility 20–45; drug-interaction crossover; detailed baseline values in source	40 g ethanol + piragliatin vs piragliatin/placebo conditions	With meals; 0–6 h	Glucose AUC ratio; hypoglycemia not primary	Special context: drug interaction
Avogaro 2004 (16) Italy	n=8 T2D; age ~58 y; BMI ~29; HbA1c ~8.2%; crossover FSIGT	Vodka providing 40 g ethanol vs water	Fasting FSIGT	Insulin sensitivity/secretion; not a clinical hypoglycemia study	Mechanistic
Rasmussen 2001 (17) Denmark	n=8; diet-treated T2D; randomized crossover clamp	0.4 g/kg ethanol vs water	Fasting hypoglycemic clamp; 7 h	Glucose recovery/glucagon; experimental clamp definition	Mechanistic counterregulation
Foot & Eastmond 1997 (18) UK	n=23 (17 men/6 women), age 43–74 y; diet-treated; parallel drug phase + crossover challenge	0.6 g/kg ethanol vs control during troglitazone/placebo treatment	Dinner; first 4 h and next morning	Weighted/fasting glucose, symptoms, urinary cortisol:creatinine; no CGM threshold	Special context: drug treatment
Gin 1992 (19) France	n=10 non-insulin-treated T2D subgroup; men; random self-control	Red wine (2 glasses) vs same lunch without wine	Lunch; postprandial curve	Curve only; numerical data incomplete; hypoglycemia definition NR	Direct postprandial; limited report
Koivisto 1993 (20) Finland	n=16 T2D; age 57 ± 2 y; BMI 27.2 ± 1.0; 14 diet/oral treated; randomized crossover	Ethanol 1 g/kg vs water/control meal	Dinner to next morning; standardized meal (45% carbohydrate, 35% fat, 20% protein)	Postprandial/fasting glucose; no participant <5 mmol/L next morning; no overnight CGM definition	Direct next-morning/overnight
Christiansen 1993 (21) Denmark	n=12; median age 55 y; BMI 26; 8 diet alone/3 oral agents/1 treatment NR; four-period crossover	Dry or sweet white wine vs water and glucose-matched conditions	Standardized light meal; 4 h	Glucose iAUC; hypoglycemia not defined	Direct postprandial
Chellingsworth 1990 (22)/UK	n=9 (8 men/1 woman); age 58.6 ± 3.9 y; BMI 28.2 ± 0.7; HbA1c 7.8 ± 0.6%; 5 diet, 3 metformin, 1 chlorpropamide; randomized-order crossover	50 mL sherry+260 mL wine: sweet (≈13.2 g sugar; 36.9 g alcohol) vs dry (<≈1.3 g sugar; 40.1 g alcohol); no alcohol-free control	Dinner (36.5 g carbohydrate) and wine at 30–90 min; snack at 150 min; 0–240 min and prebreakfast ≈14 h	Glucose curve/AUC and prebreakfast glucose; no participant reported hypoglycemic; no measurements from 4 to 14 h	Direct meal-associated active comparator; composition only
Hosaka 2008 (23)/Japan	n=6 men; age 60 ± 15 y; BMI 21.7 ± 1.8; HbA1c 6.4 ± 0.4%; diet alone; non-randomized repeated measures	Beer 800 mL (24.8 g glucose), sake 300 mL (14.7 g), or shochu 180 mL (0 g); all contained alcohol; no alcohol-free control	After 12–14 h fast; baseline, 30, 60, and 120 min	Serial blood glucose; hypoglycemia not defined; no overnight or next-morning follow-up	Fasting special-context active comparator; composition only

AUC, area under the curve; BMI, body mass index; CGM, continuous glucose monitoring; FSIGT, frequently sampled intravenous glucose tolerance test; HbA1c, glycated hemoglobin; iAUC, incremental area under the curve; NR, not reported; SI, insulin sensitivity index; T2D, type 2 diabetes.

Five direct studies evaluated wine or wine with sherry, whereas the remaining studies used isolated ethanol, spirits, beer, sake, or shochu. Beverage carbohydrate content, volume, ethanol dose, and comparators varied. Two newly included active-beverage studies lacked an alcohol-free condition and were therefore restricted to interpretation of beverage composition. Because residual carbohydrate, polyphenols, tannins, acidity, energy content, and meal context may influence the glucose response, beverage-specific findings were not generalized to ethanol as a whole. The conceptual framework for interpreting a lower next-morning glucose value is shown in **Figure 2**.

**Figure 2:**
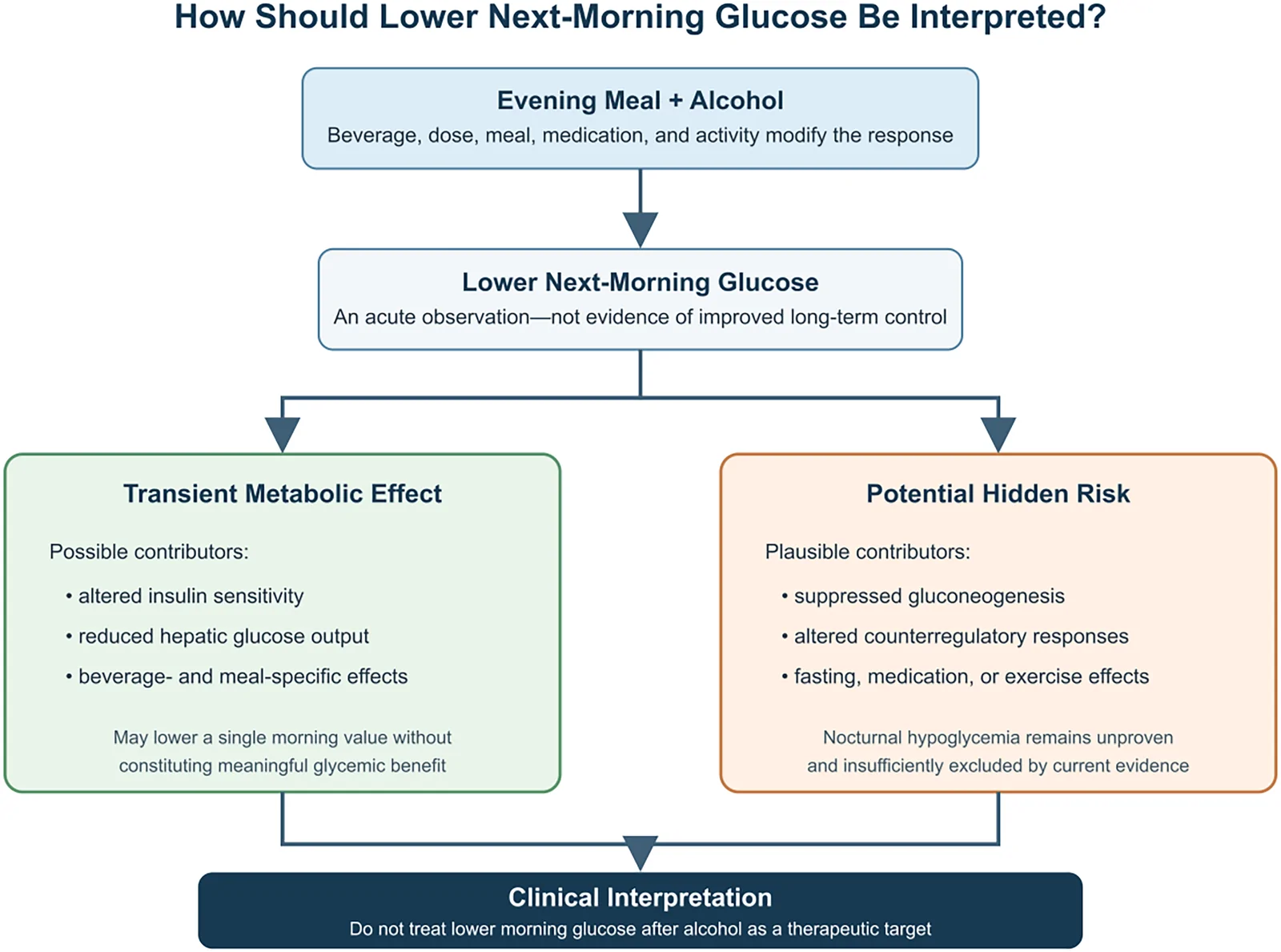
Conceptual interpretations of lower next-morning glucose after evening meal-associated alcohol consumption. The pathways are plausible alternatives and do not establish causality or documented nocturnal hypoglycemia.

### 3.3 Risk of bias

Among 11 randomized or crossover studies assessed with RoB 2, ten were judged to have some concerns and one was judged at high risk. Common concerns were incomplete reporting of allocation concealment, washout or carryover, and prespecified analyses. Chellingsworth et al. reported use of a random-number table, but concealment, washout, and a prespecified analysis plan were not described (22). The short report by Gin et al. (19) was judged at high risk because numerical outcomes and analytical details were incomplete (19). Of four studies assessed with ROBINS-I, two were judged at moderate and two at serious risk. Hosaka et al. was judged at serious risk because condition order was unreported, beverage volume and composition differed, no alcohol-free control was used, and inference relied on within-condition baseline tests without formal between-beverage comparisons (23). Objective laboratory glucose measurement generally reduced concern about outcome ascertainment; small samples were treated as imprecision rather than automatically as bias. Overall risk-of-bias judgments are summarized in **Table 3**, and domain-level judgments are shown in **Figure 3** and **4** for RoB 2 and ROBINS-I, respectively.

**Table 3:** Overall risk-of-bias judgments.

Study	Tool	Overall judgment	Principal concern
Rasmussen 1999 (8)	RoB 2 crossover	Some concerns	Randomization reporting; exercise co-intervention
Bantle 2008 (10)	RoB 2 crossover	Some concerns	Sequence/carryover reporting
Dalgaard 2004 (11)	RoB 2 crossover	Some concerns	Randomization details; meal comparability
Christiansen 1994 (13)	RoB 2 crossover	Some concerns	Sequence and washout reporting
Avogaro 2004 (16)	RoB 2 crossover	Some concerns	Randomization details; no prespecified plan
Rasmussen 2001 (17)	RoB 2 crossover	Some concerns	Randomization details
Foot 1997 (18)	RoB 2 composite	Some concerns	Complex design; missing-analysis detail
Gin 1992 (19)	RoB 2 crossover	High	Incomplete numerical and selective reporting
Koivisto 1993 (20)	RoB 2 crossover	Some concerns	Allocation concealment not described
Christiansen 1993 (21)	RoB 2 crossover	Some concerns	Carryover and selective reporting
Gin 1999 (12)	ROBINS-I	Moderate	Subgroup formation and reporting
Schaller 2010 (14)	ROBINS-I	Serious	Fixed order and period confounding
Zhi 2016 (15)	ROBINS-I	Moderate	Drug co-intervention; selective glycemic reporting
Chellingsworth 1990 (22)	RoB 2 crossover	Some concerns	Concealment, washout, and prespecified analysis not reported
Hosaka 2008 (23)	ROBINS-I	Serious	Unreported order; active comparators; co-varying beverage composition; within-baseline tests

RoB 2, revised Cochrane risk-of-bias tool for randomized trials; ROBINS-I, Risk Of Bias In Non-randomized Studies–of Interventions.

**Figure 3:**
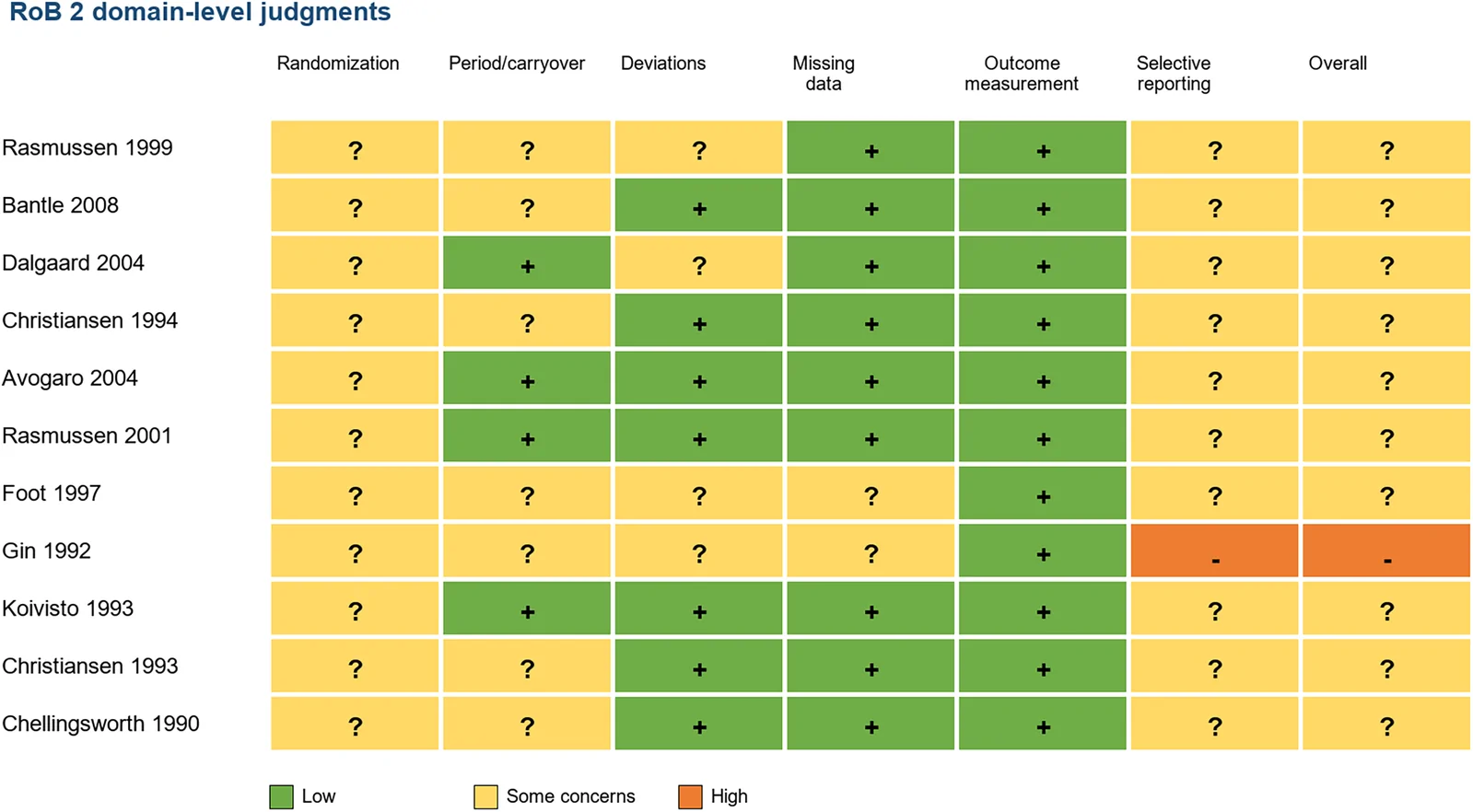
Revised Cochrane risk-of-bias tool (RoB 2) domain-level traffic-light plot for randomized and crossover studies. Green indicates low risk, yellow indicates some concerns, and orange indicates high risk.

**Figure 4:**
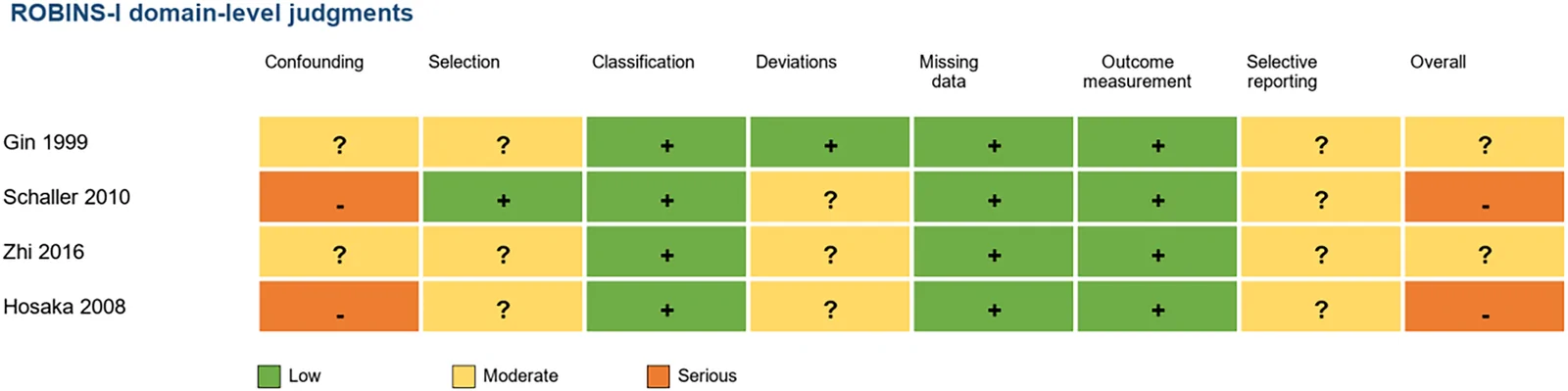
Risk Of Bias In Non-randomized Studies–of Interventions (ROBINS-I) domain-level traffic-light plot. Green indicates low risk, yellow indicates moderate risk, and orange indicates serious risk.

### 3.4 Postprandial glycemia

Among direct meal-associated studies, neither alcoholic beverages nor isolated ethanol produced a consistent postprandial glucose-lowering response. Christiansen et al. reported similar 4-h glucose iAUC after 24 g isolated ethanol and control beer (574 ± 89 vs. 554 ± 69 mmol/L·240 min; not significant) (13). A four-period study found no overall difference among water, dry wine, sweet wine, and glucose-matched wine conditions (median iAUC 497, 296, 407, and 359 mmol/L·240 min; p = 0.60) (21). Koivisto et al. found no immediate postprandial difference after dinner with ethanol (20), and Bantle et al. found no acute glucose difference between wine and grape juice during the evening/night period (10). Dalgaard et al. likewise found no clear postprandial glucose benefit when 40 g ethanol with 50 g carbohydrate was compared with an isocaloric meal containing 120 g carbohydrate, although the unequal carbohydrate composition limits causal interpretation (11).

The limited signals of attenuation were beverage specific. Gin et al. reported a lower maximum excursion with red wine than water, whereas isolated ethanol did not differ significantly from its comparator (12). An earlier red-wine report described a slower curve but lacked reliable extractable values (19). In nine participants, Chellingsworth et al. found no significant difference in evening glucose AUC between sweet and dry wine–sherry conditions (31.4 ± 2.37 vs. 29.01 ± 1.32 mmol/L·h; p = 0.25) (22). Both conditions contained alcohol, and ethanol exposure was not perfectly matched, so this comparison addresses beverage formulation rather than an effect of alcohol itself.

In a special exercise context, alcohol plus exercise reduced glucose iAUC relative to rest, but not relative to exercise alone (8). A fasting repeated-measures study of six diet-treated men found 60-min glucose values of 10.83 mmol/L after beer, 8.40 mmol/L after sake, and 5.89 mmol/L after carbohydrate-free shochu (23). Beer and sake increased glucose from their own baselines, whereas shochu did not; however, no alcohol-free condition or formal between-beverage test was reported. These special-context findings cannot establish an independent glucose-lowering effect of ethanol.

### 3.5 Overnight glucose stability and hypoglycemia

No included study documented nocturnal hypoglycemia after meal-associated exposure, but ascertainment was insufficient to rule it out. None reported nocturnal time below 3.9 mmol/L, time-to-nadir, or a prespecified CGM-based definition. Bantle et al. did not provide nocturnal nadir or time-below-range data (10), and Foot and Eastmond sampled during the first 4 h and again the next morning rather than continuously (18). Chellingsworth et al. reported no participant became hypoglycemic, but glucose was not measured between 4 h and the prebreakfast sample at approximately 14 h (22). Thus, intermittent sampling could miss brief or asymptomatic events.

### 3.6 Next-morning glucose after alcohol consumption

Only one direct study supported a lower next-morning value. In 16 participants, Koivisto et al. reported a paired reduction of 0.7 ± 0.2 mmol/L after ethanol with dinner versus control (p < 0.05); no participant had a morning glucose value below 5 mmol/L, and preceding nocturnal hypoglycemia was not measured (20). This small 1993 study had some risk-of-bias concerns, lacked overnight CGM, and has not been independently replicated. It therefore provides very-low-certainty evidence for a modest morning difference, not evidence of clinical benefit or occult hypoglycemia.

Other studies did not reproduce an alcohol-versus-no-alcohol next-morning reduction. Bantle et al. reported no significant evening or nighttime effect (10), and Foot and Eastmond reported next-morning values of 6.7 versus 6.8 mmol/L in the troglitazone group (18). Chellingsworth et al. reported prebreakfast glucose of 7.70 ± 0.77 mmol/L after sweet beverages and 7.36 ± 0.51 mmol/L after dry beverages (22); because both conditions contained alcohol and no paired inferential estimate for this time point was reported, the study neither replicates nor refutes Koivisto’s alcohol-versus-control finding.

### 3.7 Mechanistic and special-context findings

Mechanistic and special-context studies were analyzed separately from direct alcohol-versus-no-alcohol clinical evidence. Two fasting FSIGT experiments suggested increased insulin sensitivity after acute ethanol (14, 16), and a hypoglycemic clamp showed an attenuated glucagon peak but similar glucose recovery (17). Exercise, fasting beverage-composition, and drug-interaction studies showed context-dependent responses (8, 15, 18, 23). They support biological plausibility involving substrate availability, insulin action, counterregulation, and beverage carbohydrate, but do not establish a meal-associated glucose-lowering benefit or nocturnal clinical hypoglycemia. The evidence map is presented in **Table 4**, and selected harmonized quantitative findings are presented in **Table 5**.

**Table 4:** Evidence map for the clinical questions.

Clinical question	Contributing evidence	Principal finding	Certainty	Interpretation
Postprandial glycemia	8 direct meal studies; 116 participants; one active-beverage comparison	No consistent improvement; beverage composition modified responses	Very low	Beverage-specific findings do not establish an ethanol class effect
Nocturnal hypoglycemia	4 studies extending to evening/morning; 66 participants; no adequate overnight CGM dataset	No documented nocturnal event; contemporary thresholds/time-below-range not reported	Very low	Risk is neither demonstrated nor reliably excluded
Next-morning glucose	4 studies; 66 participants; only one alcohol-versus-control study (n=16) showed a significant reduction	Paired difference −0.7 ± 0.2 mmol/L in one unreplicated study	Very low	Active-comparator data do not replicate the alcohol-versus-control finding
Mechanistic/special context	3 mechanistic studies (28 participants) plus exercise, drug, and fasting beverage studies	Context-dependent insulin, counterregulatory, and beverage-carbohydrate findings	Very low/indirect	Cannot establish meal-associated overnight clinical events

CGM, continuous glucose monitoring; FSIGT, frequently sampled intravenous glucose tolerance test.

**Table 5:** Selected quantitative findings after unit harmonization.

Domain	Study (n)	Comparison	Effect estimate/result	P value/CI	Risk of bias	Interpretation
Clinical: postprandial	Christiansen 1994 (10)	24 g ethanol vs control	iAUC 574 ± 89 vs 554 ± 69 mmol/L·240 min	NS; CI NR	Some concerns	No clear difference
Clinical: postprandial	Gin 1999 (12) (30; subgroup n=10)	Red wine vs water	Peak excursion 1.8 ± 0.9 vs 2.6 ± 0.8 mmol/L	p<0.01; CI NR	Moderate	Wine-specific attenuation
Clinical: next morning	Koivisto 1993 (16)	Ethanol vs water/control meal	Paired difference −0.7 ± 0.2 mmol/L	p<0.05; CI NR	Some concerns	Isolated, unreplicated finding
Clinical: meal active comparator	Chellingsworth 1990 (9)	Sweet vs dry wine+sherry	AUC 31.4 ± 2.37 vs 29.01 ± 1.32 mmol/L·h; prebreakfast 7.70 ± 0.77 vs 7.36 ± 0.51 mmol/L	AUC p=0.25; prebreakfast p NR	Some concerns	No AUC difference; cannot estimate alcohol effect
Clinical: next morning	Foot 1997 (23)	Alcohol vs control, troglitazone group	6.7 vs 6.8 mmol/L	NS; dispersion/CI NR	Some concerns	No clear difference
Special context: exercise	Rasmussen 1999 (12)	Alcohol+exercise vs rest	iAUC 509 ± 98 vs 724 ± 109 mmol/L·240 min	p=0.03; CI NR	Some concerns	Exercise confounding
Special context: drug	Zhi 2016 (18)	Ethanol+piragliatin vs piragliatin	Glucose AUC ratio 1.13 vs 1.00	13% increase; CI NR	Moderate	Not a usual meal context
Special context: fasting beverage	Hosaka 2008 (6)	Beer vs sake vs shochu	60-min glucose 10.83 vs 8.40 vs 5.89 mmol/L	Within-baseline tests only; between-beverage p NR	Serious	Response tracked carbohydrate; no alcohol-free control
Mechanistic	Avogaro 2004 (8)	Ethanol vs water	SI 2.47 ± 0.57 vs 0.93 ± 0.26 ×10^-4^ min^-1^·μU^-1^·mL	p=0.009; CI NR	Some concerns	Fasting FSIGT; indirect
Mechanistic	Rasmussen 2001 (8)	Ethanol vs water during clamp	Glucagon peak 155 ± 12 vs 200 ± 17 pg/mL	p=0.038; CI NR	Some concerns	Recovery unchanged; indirect

AUC, area under the curve; CI, confidence interval; FSIGT, frequently sampled intravenous glucose tolerance test; iAUC, incremental area under the curve; NR, not reported; NS, not statistically significant; SI, insulin sensitivity index.

## 4 Discussion

Across 15 small studies, direct clinical evidence did not show a reproducible acute glucose-lowering effect of meal-associated alcoholic beverages or ethanol. The two Embase-identified active-beverage comparisons sharpen the interpretation rather than changing it: beverage carbohydrate and formulation affect early glucose responses, but comparisons in which every condition contains alcohol cannot estimate whether alcohol lowers glucose. The central next-morning observation remains confined to one unreplicated alcohol-versus-control study of 16 participants, while nocturnal hypoglycemia was neither demonstrated nor adequately assessed.

### 4.1 Postprandial glycemic responses and their interpretation

Postprandial findings differed by exposure. Isolated ethanol generally did not produce a dependable reduction, whereas selected red-wine experiments reported smaller excursions. The new evidence reinforces why wine, beer, sake, shochu, and ethanol cannot be treated as interchangeable. In Chellingsworth et al., a roughly tenfold difference in beverage sugar content produced only a nonsignificant AUC difference under a high-fiber meal, but the study was underpowered and alcohol doses also differed slightly (22). In Hosaka et al., fasting glucose excursions broadly followed beverage glucose content, yet volume, ethanol concentration, and carbohydrate were co-varied and no between-condition test was performed (23). These data point to beverage matrix and carbohydrate as modifiers, not to a therapeutic effect of ethanol.

Exercise is another important modifier. In Rasmussen et al., alcohol combined with exercise reduced incremental glucose exposure relative to rest, but not relative to exercise alone (8). Interpreting this result as an alcohol effect would overstate the evidence. Likewise, drug-interaction and special-context studies provide mechanistic or safety information but should not be treated as evidence for a usual meal-associated drinking pattern.

### 4.2 Overnight glycemia and hypoglycemia

Overnight safety remains unresolved rather than established as either harm or safety. Ethanol can inhibit gluconeogenesis and influence counterregulatory responses (1, 17), but the eligible studies did not observe glucose continuously with contemporary CGM definitions. The absence of recorded events under intermittent sampling cannot exclude short, asymptomatic episodes; equally, mechanistic plausibility cannot be used as proof that such episodes occurred.

There is a physiological basis for caution. Ethanol inhibits gluconeogenesis (1), potentially reducing endogenous glucose availability several hours after dinner. It can also attenuate hormonal responses to falling glucose. During experimental hypoglycemia, glucagon, growth hormone, and cortisol responses were lower after ethanol, although glucose recovery was preserved under those controlled conditions (17). Insulin or sulfonylurea treatment, fasting, prolonged exercise, depleted glycogen stores, or larger alcohol doses could make these effects more consequential.

This uncertainty is greatest for people using insulin or insulin secretagogues, who were scarcely represented. Findings from predominantly diet- or oral-agent-treated participants should not be extrapolated to these higher-risk groups. Future trials should stratify therapy and monitor glucose from before dinner through breakfast, reporting time below 3.9 mmol/L, time below 3.0 mmol/L, nocturnal nadir, symptoms, and rescue treatment.

### 4.3 Interpreting next-morning fasting glucose

The next-morning question still rests on one small, older alcohol-versus-control study: a 0.7 mmol/L paired reduction was reported after ethanol with dinner, but morning values were not hypoglycemic and no preceding overnight event was documented (20). The sweet-versus-dry study reported similar prebreakfast values after two alcohol-containing conditions and therefore does not provide independent replication (22). Incomplete allocation reporting, absence of overnight CGM, active comparators in the newer evidence, and lack of independent alcohol-free replication keep certainty very low.

A single lower fasting value provides no information about overnight exposure, glycemic variability, HbA1c, or complications and should not be interpreted as improved diabetes control. The evidence does not support deliberate alcohol use to lower morning glucose. For people who choose to drink, clinical advice should consider beverage type and amount, food intake, physical activity, glucose-lowering therapy, comorbidity, and prior hypoglycemia, without extrapolating the low-risk study populations to insulin- or secretagogue-treated patients (3, 17).

### 4.4 Broader clinical implications of long-term alcohol exposure

This review did not evaluate the long-term effects of drinking, and its acute findings should not be extrapolated to HbA1c, complication risk, or sustained diabetes control. Regular alcohol consumption also adds energy, may add carbohydrate depending on the beverage, and can interact with eating patterns and glucose-lowering medication. These considerations change the clinical balance even when a short-term glucose value appears favorable.

For people using insulin or insulin secretagogues, alcohol may add to delayed hypoglycemia risk when food intake is inadequate or exercise is prolonged; intoxication can also hinder symptom recognition and self-management. Liver disease, pancreatitis, neuropathy, and adverse cardiovascular risk profiles provide further reasons for caution, although these outcomes were outside the scope of the included acute studies and cannot be quantified here (3).

Alcohol should not be recommended as a glucose-lowering intervention. Clinical advice should reflect the amount and pattern of use, medication regimen, food intake, physical activity, comorbidities, and the person’s risk of hypoglycemia.

### 4.5 Limitations

The evidence base was limited by small samples, short observation, older studies, heterogeneous beverages and ethanol doses, inconsistent meals and comparators, and incompatible outcome times and statistics. Two included studies used only active alcohol-containing comparators; they inform beverage composition but cannot estimate alcohol versus no alcohol. These differences, together with missing paired-condition variances, precluded an interpretable meta-analysis. No eligible study provided contemporary overnight CGM metrics, and most participants were treated with diet or oral agents, limiting inference for insulin- or insulin-secretagogue-treated populations.

Four result-bearing reports remained unavailable despite repeated bibliographic and public searches and author contact; no response had been received by 31 August 2026. Publication bias could not be assessed formally. PROSPERO registration occurred after review initiation, and the active-comparator criterion was specified after registration. Although active-comparator evidence was kept analytically separate, this timing introduces a potential *post hoc* selection concern. No third reviewer was available for unresolved adjudication, although two reviewers independently completed screening, extraction, and risk-of-bias assessment.

Future studies should use standardized evening meal and alcohol protocols, stratify participants by glucose-lowering therapy, and apply CGM from before dinner through the following breakfast. Reporting time below range, nocturnal nadir, time-to-nadir, next-morning glucose, postprandial area under the curve, and paired-difference variances would permit a more definitive assessment of safety and clinical relevance.

## 5 Conclusion

Very-low-certainty evidence from 15 small, heterogeneous studies does not show that meal-associated alcoholic beverages or ethanol consistently improve postprandial glycemia. The added active-beverage comparisons strengthen the conclusion that carbohydrate content and beverage matrix shape early glucose responses, but they do not estimate alcohol versus abstinence. The central next-morning observation remains one unreplicated crossover finding and does not establish better diabetes control. Current evidence neither demonstrates that a lower morning value is caused by nocturnal hypoglycemia nor reliably excludes brief or asymptomatic events, because adequate overnight CGM evidence and higher-risk insulin- or secretagogue-treated populations are lacking. Alcohol should not be used as a glucose-lowering strategy.

## Data Availability

The original contributions presented in the study are included in the article/**Supplementary Material**. Further inquiries can be directed to the corresponding author/s.
