# Supplementary material for: Acute glycemic responses to meal-associated alcoholic beverages and ethanol in adults with type 2 diabetes: a systematic review
Source: Front Endocrinol (Lausanne). 2026 Sep 29;17:1967515. doi: 10.3389/fendo.2026.1967515 (PMC13658088; doi:10.3389/fendo.2026.1967515)
Supplement: Supplementary file 1 [file SupplementaryFile1.docx]

Supplementary material

# Contents

Supplementary Table S1. Database search strategies and supplementary-search methods.

Supplementary Table S2. Study-level data extraction.

Supplementary Table S3. Selected glucose outcome data after unit harmonization.

Supplementary Table S4. Risk-of-bias judgments.

Supplementary Table S5. Certainty of evidence (GRADE considerations).

Supplementary Table S6. Full-text exclusions and unavailable reports.

Supplementary Table S7. Structured effect-direction and heterogeneity matrix.

# Supplementary Table S1. Database search strategies and supplementary-search methods.

PubMed, Web of Science Core Collection, the Cochrane Library, four EBSCOhost databases—MEDLINE with Full Text, SPORTDiscus with Full Text, eBook Collection (EBSCOhost), and OpenDissertations—and Embase.com were searched from inception to 28 August 2026. The searches yielded 489 PubMed records, 874 Web of Science Core Collection records, 402 Cochrane Library records, 159 EBSCOhost records, 3,257 Embase article records, and 126 Embase Clinical Trials register records. Register records were screened separately and were not treated as result-bearing studies unless posted results or a linked publication was available. Acute comparisons between two alcohol-containing beverages were eligible when they provided glycemic data in adults with type 2 diabetes; such evidence was classified as active-comparator composition evidence and was not interpreted as alcohol versus abstinence. Reference lists were checked. Four result-bearing reports remained unavailable after repeated searches by title, author, identifier, and publisher link. Corresponding authors were contacted by email, but no response had been received by 31 August 2026. For active-comparator studies, effect direction referred only to differences between the alcohol-containing beverage conditions and was not interpreted as an alcohol-versus-abstinence effect.

PubMed. Database searched from inception to 28 August 2026; no publication-year restriction; records exported: n = 489.

(Diabetes Mellitus, Adult-Onset[Title/Abstract] OR Adult-Onset Diabetes Mellitus[Title/Abstract] OR Diabetes Mellitus, Adult Onset[Title/Abstract] OR Diabetes Mellitus, Ketosis-Resistant[Title/Abstract] OR Diabetes Mellitus, Ketosis Resistant[Title/Abstract] OR Ketosis-Resistant Diabetes Mellitus[Title/Abstract] OR NIDDM[Title/Abstract] OR Diabetes Mellitus, Maturity-Onset[Title/Abstract] OR Diabetes Mellitus, Maturity Onset[Title/Abstract] OR Diabetes Mellitus, Non Insulin Dependent[Title/Abstract] OR Diabetes Mellitus, Non-Insulin-Dependent[Title/Abstract] OR Non-Insulin-Dependent Diabetes Mellitus[Title/Abstract] OR Diabetes Mellitus, Noninsulin Dependent[Title/Abstract] OR Diabetes Mellitus, Noninsulin-Dependent[Title/Abstract] OR Diabetes Mellitus, Slow-Onset[Title/Abstract] OR Diabetes Mellitus, Slow Onset[Title/Abstract] OR Slow-Onset Diabetes Mellitus[Title/Abstract] OR Diabetes Mellitus, Stable[Title/Abstract] OR Stable Diabetes Mellitus[Title/Abstract] OR Diabetes Mellitus, Type II[Title/Abstract] OR Maturity-Onset Diabetes[Title/Abstract] OR Diabetes, Maturity-Onset[Title/Abstract] OR Maturity Onset Diabetes[Title/Abstract] OR Maturity-Onset Diabetes Mellitus[Title/Abstract] OR Maturity Onset Diabetes Mellitus[Title/Abstract] OR Noninsulin-Dependent Diabetes Mellitus[Title/Abstract] OR Noninsulin Dependent Diabetes Mellitus[Title/Abstract] OR Type 2 Diabetes[Title/Abstract] OR Diabetes, Type 2[Title/Abstract] OR Type 2 Diabetes Mellitus[Title/Abstract] OR T2D[Title/Abstract] OR T2DM[Title/Abstract] OR Type II Diabetes[Title/Abstract] OR Type II Diabetes Mellitus[Title/Abstract] OR Adult Onset Diabetes[Title/Abstract] OR Adult Onset Diabetes Mellitus[Title/Abstract] OR Non Insulin Dependent Diabetes[Title/Abstract] OR Non Insulin Dependent Diabetes Mellitus[Title/Abstract]) AND (Alcohol, Ethyl[Title/Abstract] OR Ethyl Alcohol[Title/Abstract] OR Grain Alcohol[Title/Abstract] OR Alcohol, Grain[Title/Abstract] OR Absolute Alcohol[Title/Abstract] OR Alcohol, Absolute[Title/Abstract] OR Alcoholic Beverage[Title/Abstract] OR Alcoholic Beverages[Title/Abstract] OR Alcohol Drinking[Title/Abstract] OR Alcohol Consumption[Title/Abstract] OR Alcohol Intake[Title/Abstract] OR Alcohol Ingestion[Title/Abstract] OR Ethanol Consumption[Title/Abstract] OR Ethanol Intake[Title/Abstract] OR Ethanol Ingestion[Title/Abstract] OR Wine[Title/Abstract] OR Red Wine[Title/Abstract] OR White Wine[Title/Abstract] OR Beer[Title/Abstract] OR Alcoholic Beer[Title/Abstract] OR Spirits[Title/Abstract] OR Distilled Spirits[Title/Abstract] OR Liquor[Title/Abstract] OR Distilled Liquor[Title/Abstract] OR Whisky[Title/Abstract] OR Whiskey[Title/Abstract] OR Vodka[Title/Abstract] OR Gin[Title/Abstract] OR Rum[Title/Abstract] OR Brandy[Title/Abstract] OR Baijiu[Title/Abstract] OR Chinese Baijiu[Title/Abstract] OR Chinese Liquor[Title/Abstract] OR Chinese Distilled Liquor[Title/Abstract] OR Chinese Spirits[Title/Abstract] OR Chinese Distilled Spirits[Title/Abstract] OR Chinese White Spirit[Title/Abstract]) AND (Glucose, Blood[Title/Abstract] OR Blood Sugar[Title/Abstract] OR Sugar, Blood[Title/Abstract] OR Blood Glucose[Title/Abstract] OR Plasma Glucose[Title/Abstract] OR Serum Glucose[Title/Abstract] OR Glycemia[Title/Abstract] OR Glycaemia[Title/Abstract] OR Glycemic Response[Title/Abstract] OR Glycaemic Response[Title/Abstract] OR Glucose Response[Title/Abstract] OR Glucose Homeostasis[Title/Abstract] OR Postprandial Glucose[Title/Abstract] OR Postprandial Glycemia[Title/Abstract] OR Postprandial Glycaemia[Title/Abstract] OR Fasting Blood Glucose[Title/Abstract] OR Fasting Plasma Glucose[Title/Abstract] OR Fasting Glucose[Title/Abstract] OR Morning Glucose[Title/Abstract] OR Next-Morning Glucose[Title/Abstract] OR Overnight Glucose[Title/Abstract] OR Nocturnal Glucose[Title/Abstract] OR Hypoglycemia[Title/Abstract] OR Hypoglycaemia[Title/Abstract] OR Nocturnal Hypoglycemia[Title/Abstract] OR Nocturnal Hypoglycaemia[Title/Abstract] OR Delayed Hypoglycemia[Title/Abstract] OR Delayed Hypoglycaemia[Title/Abstract] OR Glucose Area Under the Curve[Title/Abstract] OR Glucose AUC[Title/Abstract] OR Glycemic Variability[Title/Abstract] OR Glycaemic Variability[Title/Abstract] OR Continuous Glucose Monitoring[Title/Abstract] OR CGM[Title/Abstract] OR Time in Range[Title/Abstract] OR Time Below Range[Title/Abstract] OR TIR[Title/Abstract] OR TBR[Title/Abstract])

Web of Science Core Collection. Database searched from inception to 28 August 2026; no publication-year restriction; records exported: n = 874.

(TS=(Diabetes Mellitus, Adult-Onset) OR TS=(Adult-Onset Diabetes Mellitus) OR TS=(Diabetes Mellitus, Adult Onset) OR TS=(Diabetes Mellitus, Ketosis-Resistant) OR TS=(Diabetes Mellitus, Ketosis Resistant) OR TS=(Ketosis-Resistant Diabetes Mellitus) OR TS=(NIDDM) OR TS=(Diabetes Mellitus, Maturity-Onset) OR TS=(Diabetes Mellitus, Maturity Onset) OR TS=(Diabetes Mellitus, Non Insulin Dependent) OR TS=(Diabetes Mellitus, Non-Insulin-Dependent) OR TS=(Non-Insulin-Dependent Diabetes Mellitus) OR TS=(Diabetes Mellitus, Noninsulin Dependent) OR TS=(Diabetes Mellitus, Noninsulin-Dependent) OR TS=(Diabetes Mellitus, Slow-Onset) OR TS=(Diabetes Mellitus, Slow Onset) OR TS=(Slow-Onset Diabetes Mellitus) OR TS=(Diabetes Mellitus, Stable) OR TS=(Stable Diabetes Mellitus) OR TS=(Diabetes Mellitus, Type II) OR TS=(Maturity-Onset Diabetes) OR TS=(Diabetes, Maturity-Onset) OR TS=(Maturity Onset Diabetes) OR TS=(Maturity-Onset Diabetes Mellitus) OR TS=(Maturity Onset Diabetes Mellitus) OR TS=(Noninsulin-Dependent Diabetes Mellitus) OR TS=(Noninsulin Dependent Diabetes Mellitus) OR TS=(Type 2 Diabetes) OR TS=(Diabetes, Type 2) OR TS=(Type 2 Diabetes Mellitus) OR TS=(T2D) OR TS=(T2DM) OR TS=(Type II Diabetes) OR TS=(Type II Diabetes Mellitus) OR TS=(Adult Onset Diabetes) OR TS=(Adult Onset Diabetes Mellitus) OR TS=(Non Insulin Dependent Diabetes) OR TS=(Non Insulin Dependent Diabetes Mellitus)) AND (TS=(Alcohol, Ethyl) OR TS=(Ethyl Alcohol) OR TS=(Grain Alcohol) OR TS=(Alcohol, Grain) OR TS=(Absolute Alcohol) OR TS=(Alcohol, Absolute) OR TS=(Alcoholic Beverage) OR TS=(Alcoholic Beverages) OR TS=(Alcohol Drinking) OR TS=(Alcohol Consumption) OR TS=(Alcohol Intake) OR TS=(Alcohol Ingestion) OR TS=(Ethanol Consumption) OR TS=(Ethanol Intake) OR TS=(Ethanol Ingestion) OR TS=(Wine) OR TS=(Red Wine) OR TS=(White Wine) OR TS=(Beer) OR TS=(Alcoholic Beer) OR TS=(Spirits) OR TS=(Distilled Spirits) OR TS=(Liquor) OR TS=(Distilled Liquor) OR TS=(Whisky) OR TS=(Whiskey) OR TS=(Vodka) OR TS=(Gin) OR TS=(Rum) OR TS=(Brandy) OR TS=(Baijiu) OR TS=(Chinese Baijiu) OR TS=(Chinese Liquor) OR TS=(Chinese Distilled Liquor) OR TS=(Chinese Spirits) OR TS=(Chinese Distilled Spirits) OR TS=(Chinese White Spirit)) AND (TS=(Glucose, Blood) OR TS=(Blood Sugar) OR TS=(Sugar, Blood) OR TS=(Blood Glucose) OR TS=(Plasma Glucose) OR TS=(Serum Glucose) OR TS=(Glycemia) OR TS=(Glycaemia) OR TS=(Glycemic Response) OR TS=(Glycaemic Response) OR TS=(Glucose Response) OR TS=(Glucose Homeostasis) OR TS=(Postprandial Glucose) OR TS=(Postprandial Glycemia) OR TS=(Postprandial Glycaemia) OR TS=(Fasting Blood Glucose) OR TS=(Fasting Plasma Glucose) OR TS=(Fasting Glucose) OR TS=(Morning Glucose) OR TS=(Next-Morning Glucose) OR TS=(Overnight Glucose) OR TS=(Nocturnal Glucose) OR TS=(Hypoglycemia) OR TS=(Hypoglycaemia) OR TS=(Nocturnal Hypoglycemia) OR TS=(Nocturnal Hypoglycaemia) OR TS=(Delayed Hypoglycemia) OR TS=(Delayed Hypoglycaemia) OR TS=(Glucose Area Under the Curve) OR TS=(Glucose AUC) OR TS=(Glycemic Variability) OR TS=(Glycaemic Variability) OR TS=(Continuous Glucose Monitoring) OR TS=(CGM) OR TS=(Time in Range) OR TS=(Time Below Range) OR TS=(TIR) OR TS=(TBR))

Cochrane Library. Database searched from inception to 28 August 2026; no publication-year restriction; records exported: n = 402.

((Diabetes Mellitus, Adult-Onset):ti,ab,kw OR (Adult-Onset Diabetes Mellitus):ti,ab,kw OR (Diabetes Mellitus, Adult Onset):ti,ab,kw OR (Diabetes Mellitus, Ketosis-Resistant):ti,ab,kw OR (Diabetes Mellitus, Ketosis Resistant):ti,ab,kw OR (Ketosis-Resistant Diabetes Mellitus):ti,ab,kw OR (NIDDM):ti,ab,kw OR (Diabetes Mellitus, Maturity-Onset):ti,ab,kw OR (Diabetes Mellitus, Maturity Onset):ti,ab,kw OR (Diabetes Mellitus, Non Insulin Dependent):ti,ab,kw OR (Diabetes Mellitus, Non-Insulin-Dependent):ti,ab,kw OR (Non-Insulin-Dependent Diabetes Mellitus):ti,ab,kw OR (Diabetes Mellitus, Noninsulin Dependent):ti,ab,kw OR (Diabetes Mellitus, Noninsulin-Dependent):ti,ab,kw OR (Diabetes Mellitus, Slow-Onset):ti,ab,kw OR (Diabetes Mellitus, Slow Onset):ti,ab,kw OR (Slow-Onset Diabetes Mellitus):ti,ab,kw OR (Diabetes Mellitus, Stable):ti,ab,kw OR (Stable Diabetes Mellitus):ti,ab,kw OR (Diabetes Mellitus, Type II):ti,ab,kw OR (Maturity-Onset Diabetes):ti,ab,kw OR (Diabetes, Maturity-Onset):ti,ab,kw OR (Maturity Onset Diabetes):ti,ab,kw OR (Maturity-Onset Diabetes Mellitus):ti,ab,kw OR (Maturity Onset Diabetes Mellitus):ti,ab,kw OR (Noninsulin-Dependent Diabetes Mellitus):ti,ab,kw OR (Noninsulin Dependent Diabetes Mellitus):ti,ab,kw OR (Type 2 Diabetes):ti,ab,kw OR (Diabetes, Type 2):ti,ab,kw OR (Type 2 Diabetes Mellitus):ti,ab,kw OR (T2D):ti,ab,kw OR (T2DM):ti,ab,kw OR (Type II Diabetes):ti,ab,kw OR (Type II Diabetes Mellitus):ti,ab,kw OR (Adult Onset Diabetes):ti,ab,kw OR (Adult Onset Diabetes Mellitus):ti,ab,kw OR (Non Insulin Dependent Diabetes):ti,ab,kw OR (Non Insulin Dependent Diabetes Mellitus):ti,ab,kw) AND ((Alcohol, Ethyl):ti,ab,kw OR (Ethyl Alcohol):ti,ab,kw OR (Grain Alcohol):ti,ab,kw OR (Alcohol, Grain):ti,ab,kw OR (Absolute Alcohol):ti,ab,kw OR (Alcohol, Absolute):ti,ab,kw OR (Alcoholic Beverage):ti,ab,kw OR (Alcoholic Beverages):ti,ab,kw OR (Alcohol Drinking):ti,ab,kw OR (Alcohol Consumption):ti,ab,kw OR (Alcohol Intake):ti,ab,kw OR (Alcohol Ingestion):ti,ab,kw OR (Ethanol Consumption):ti,ab,kw OR (Ethanol Intake):ti,ab,kw OR (Ethanol Ingestion):ti,ab,kw OR (Wine):ti,ab,kw OR (Red Wine):ti,ab,kw OR (White Wine):ti,ab,kw OR (Beer):ti,ab,kw OR (Alcoholic Beer):ti,ab,kw OR (Spirits):ti,ab,kw OR (Distilled Spirits):ti,ab,kw OR (Liquor):ti,ab,kw OR (Distilled Liquor):ti,ab,kw OR (Whisky):ti,ab,kw OR (Whiskey):ti,ab,kw OR (Vodka):ti,ab,kw OR (Gin):ti,ab,kw OR (Rum):ti,ab,kw OR (Brandy):ti,ab,kw OR (Baijiu):ti,ab,kw OR (Chinese Baijiu):ti,ab,kw OR (Chinese Liquor):ti,ab,kw OR (Chinese Distilled Liquor):ti,ab,kw OR (Chinese Spirits):ti,ab,kw OR (Chinese Distilled Spirits):ti,ab,kw OR (Chinese White Spirit):ti,ab,kw) AND ((Glucose, Blood):ti,ab,kw OR (Blood Sugar):ti,ab,kw OR (Sugar, Blood):ti,ab,kw OR (Blood Glucose):ti,ab,kw OR (Plasma Glucose):ti,ab,kw OR (Serum Glucose):ti,ab,kw OR (Glycemia):ti,ab,kw OR (Glycaemia):ti,ab,kw OR (Glycemic Response):ti,ab,kw OR (Glycaemic Response):ti,ab,kw OR (Glucose Response):ti,ab,kw OR (Glucose Homeostasis):ti,ab,kw OR (Postprandial Glucose):ti,ab,kw OR (Postprandial Glycemia):ti,ab,kw OR (Postprandial Glycaemia):ti,ab,kw OR (Fasting Blood Glucose):ti,ab,kw OR (Fasting Plasma Glucose):ti,ab,kw OR (Fasting Glucose):ti,ab,kw OR (Morning Glucose):ti,ab,kw OR (Next-Morning Glucose):ti,ab,kw OR (Overnight Glucose):ti,ab,kw OR (Nocturnal Glucose):ti,ab,kw OR (Hypoglycemia):ti,ab,kw OR (Hypoglycaemia):ti,ab,kw OR (Nocturnal Hypoglycemia):ti,ab,kw OR (Nocturnal Hypoglycaemia):ti,ab,kw OR (Delayed Hypoglycemia):ti,ab,kw OR (Delayed Hypoglycaemia):ti,ab,kw OR (Glucose Area Under the Curve):ti,ab,kw OR (Glucose AUC):ti,ab,kw OR (Glycemic Variability):ti,ab,kw OR (Glycaemic Variability):ti,ab,kw OR (Continuous Glucose Monitoring):ti,ab,kw OR (CGM):ti,ab,kw OR (Time in Range):ti,ab,kw OR (Time Below Range):ti,ab,kw OR (TIR):ti,ab,kw OR (TBR):ti,ab,kw)

EBSCOhost: MEDLINE with Full Text, SPORTDiscus with Full Text, eBook Collection (EBSCOhost), and OpenDissertations. Searched from inception to 28 August 2026; no publication-year restriction; combined records exported: n = 159.

S1: SU (Diabetes Mellitus, Adult-Onset) OR SU (Adult-Onset Diabetes Mellitus) OR SU (Diabetes Mellitus, Adult Onset) OR SU (Diabetes Mellitus, Ketosis-Resistant) OR SU (Diabetes Mellitus, Ketosis Resistant) OR SU (Ketosis-Resistant Diabetes Mellitus) OR SU (NIDDM) OR SU (Diabetes Mellitus, Maturity-Onset) OR SU (Diabetes Mellitus, Maturity Onset) OR SU (Diabetes Mellitus, Non Insulin Dependent) OR SU (Diabetes Mellitus, Non-Insulin-Dependent) OR SU (Non-Insulin-Dependent Diabetes Mellitus) OR SU (Diabetes Mellitus, Noninsulin Dependent) OR SU (Diabetes Mellitus, Noninsulin-Dependent) OR SU (Diabetes Mellitus, Slow-Onset) OR SU (Diabetes Mellitus, Slow Onset) OR SU (Slow-Onset Diabetes Mellitus) OR SU (Diabetes Mellitus, Stable) OR SU (Stable Diabetes Mellitus) OR SU (Diabetes Mellitus, Type II) OR SU (Maturity-Onset Diabetes) OR SU (Diabetes, Maturity-Onset) OR SU (Maturity Onset Diabetes) OR SU (Maturity-Onset Diabetes Mellitus) OR SU (Maturity Onset Diabetes Mellitus) OR SU (Noninsulin-Dependent Diabetes Mellitus) OR SU (Noninsulin Dependent Diabetes Mellitus) OR SU (Type 2 Diabetes) OR SU (Diabetes, Type 2) OR SU (Type 2 Diabetes Mellitus) OR SU (T2D) OR SU (T2DM) OR SU (Type II Diabetes) OR SU (Type II Diabetes Mellitus) OR SU (Adult Onset Diabetes) OR SU (Adult Onset Diabetes Mellitus) OR SU (Non Insulin Dependent Diabetes) OR SU (Non Insulin Dependent Diabetes Mellitus)
S2: SU (Alcohol, Ethyl) OR SU (Ethyl Alcohol) OR SU (Grain Alcohol) OR SU (Alcohol, Grain) OR SU (Absolute Alcohol) OR SU (Alcohol, Absolute) OR SU (Alcoholic Beverage) OR SU (Alcoholic Beverages) OR SU (Alcohol Drinking) OR SU (Alcohol Consumption) OR SU (Alcohol Intake) OR SU (Alcohol Ingestion) OR SU (Ethanol Consumption) OR SU (Ethanol Intake) OR SU (Ethanol Ingestion) OR SU (Wine) OR SU (Red Wine) OR SU (White Wine) OR SU (Beer) OR SU (Alcoholic Beer) OR SU (Spirits) OR SU (Distilled Spirits) OR SU (Liquor) OR SU (Distilled Liquor) OR SU (Whisky) OR SU (Whiskey) OR SU (Vodka) OR SU (Gin) OR SU (Rum) OR SU (Brandy) OR SU (Baijiu) OR SU (Chinese Baijiu) OR SU (Chinese Liquor) OR SU (Chinese Distilled Liquor) OR SU (Chinese Spirits) OR SU (Chinese Distilled Spirits) OR SU (Chinese White Spirit)
S3: SU (Glucose, Blood) OR SU (Blood Sugar) OR SU (Sugar, Blood) OR SU (Blood Glucose) OR SU (Plasma Glucose) OR SU (Serum Glucose) OR SU (Glycemia) OR SU (Glycaemia) OR SU (Glycemic Response) OR SU (Glycaemic Response) OR SU (Glucose Response) OR SU (Glucose Homeostasis) OR SU (Postprandial Glucose) OR SU (Postprandial Glycemia) OR SU (Postprandial Glycaemia) OR SU (Fasting Blood Glucose) OR SU (Fasting Plasma Glucose) OR SU (Fasting Glucose) OR SU (Morning Glucose) OR SU (Next-Morning Glucose) OR SU (Overnight Glucose) OR SU (Nocturnal Glucose) OR SU (Hypoglycemia) OR SU (Hypoglycaemia) OR SU (Nocturnal Hypoglycemia) OR SU (Nocturnal Hypoglycaemia) OR SU (Delayed Hypoglycemia) OR SU (Delayed Hypoglycaemia) OR SU (Glucose Area Under the Curve) OR SU (Glucose AUC) OR SU (Glycemic Variability) OR SU (Glycaemic Variability) OR SU (Continuous Glucose Monitoring) OR SU (CGM) OR SU (Time in Range) OR SU (Time Below Range) OR SU (TIR) OR SU (TBR)
Final combination: S1 AND S2 AND S3

**Embase.com. Searched from inception on 28 August 2026; no publication-year restriction; article records exported: n = 3,257; Embase Clinical Trials register records exported separately: n = 126.**

('non insulin dependent diabetes mellitus'/exp OR 'diabetes mellitus, adult-onset':ti,ab OR 'adult-onset diabetes mellitus':ti,ab OR 'diabetes mellitus, adult onset':ti,ab OR 'diabetes mellitus, ketosis-resistant':ti,ab OR 'diabetes mellitus, ketosis resistant':ti,ab OR 'ketosis-resistant diabetes mellitus':ti,ab OR 'niddm':ti,ab OR 'diabetes mellitus, maturity-onset':ti,ab OR 'diabetes mellitus, maturity onset':ti,ab OR 'diabetes mellitus, non insulin dependent':ti,ab OR 'diabetes mellitus, non-insulin-dependent':ti,ab OR 'non-insulin-dependent diabetes mellitus':ti,ab OR 'diabetes mellitus, noninsulin dependent':ti,ab OR 'diabetes mellitus, noninsulin-dependent':ti,ab OR 'diabetes mellitus, slow-onset':ti,ab OR 'diabetes mellitus, slow onset':ti,ab OR 'slow-onset diabetes mellitus':ti,ab OR 'diabetes mellitus, stable':ti,ab OR 'stable diabetes mellitus':ti,ab OR 'diabetes mellitus, type ii':ti,ab OR 'maturity-onset diabetes':ti,ab OR 'diabetes, maturity-onset':ti,ab OR 'maturity onset diabetes':ti,ab OR 'maturity-onset diabetes mellitus':ti,ab OR 'maturity onset diabetes mellitus':ti,ab OR 'noninsulin-dependent diabetes mellitus':ti,ab OR 'noninsulin dependent diabetes mellitus':ti,ab OR 'type 2 diabetes':ti,ab OR 'diabetes, type 2':ti,ab OR 'type 2 diabetes mellitus':ti,ab OR 't2d':ti,ab OR 't2dm':ti,ab OR 'type ii diabetes':ti,ab OR 'type ii diabetes mellitus':ti,ab OR 'adult onset diabetes':ti,ab OR 'adult onset diabetes mellitus':ti,ab OR 'non insulin dependent diabetes':ti,ab OR 'non insulin dependent diabetes mellitus':ti,ab)
AND
('alcohol consumption'/exp OR 'alcohol, ethyl':ti,ab OR 'ethyl alcohol':ti,ab OR 'grain alcohol':ti,ab OR 'alcohol, grain':ti,ab OR 'absolute alcohol':ti,ab OR 'alcohol, absolute':ti,ab OR 'alcoholic beverage':ti,ab OR 'alcoholic beverages':ti,ab OR 'alcohol drinking':ti,ab OR 'alcohol consumption':ti,ab OR 'alcohol intake':ti,ab OR 'alcohol ingestion':ti,ab OR 'ethanol consumption':ti,ab OR 'ethanol intake':ti,ab OR 'ethanol ingestion':ti,ab OR 'wine':ti,ab OR 'red wine':ti,ab OR 'white wine':ti,ab OR 'beer':ti,ab OR 'alcoholic beer':ti,ab OR 'spirits':ti,ab OR 'distilled spirits':ti,ab OR 'liquor':ti,ab OR 'distilled liquor':ti,ab OR 'whisky':ti,ab OR 'whiskey':ti,ab OR 'vodka':ti,ab OR 'gin':ti,ab OR 'rum':ti,ab OR 'brandy':ti,ab OR 'baijiu':ti,ab OR 'chinese baijiu':ti,ab OR 'chinese liquor':ti,ab OR 'chinese distilled liquor':ti,ab OR 'chinese spirits':ti,ab OR 'chinese distilled spirits':ti,ab OR 'chinese white spirit':ti,ab)
AND
('blood glucose'/exp OR 'glucose, blood':ti,ab OR 'blood sugar':ti,ab OR 'sugar, blood':ti,ab OR 'blood glucose':ti,ab OR 'plasma glucose':ti,ab OR 'serum glucose':ti,ab OR 'glycemia':ti,ab OR 'glycaemia':ti,ab OR 'glycemic response':ti,ab OR 'glycaemic response':ti,ab OR 'glucose response':ti,ab OR 'glucose homeostasis':ti,ab OR 'postprandial glucose':ti,ab OR 'postprandial glycemia':ti,ab OR 'postprandial glycaemia':ti,ab OR 'fasting blood glucose':ti,ab OR 'fasting plasma glucose':ti,ab OR 'fasting glucose':ti,ab OR 'morning glucose':ti,ab OR 'next-morning glucose':ti,ab OR 'overnight glucose':ti,ab OR 'nocturnal glucose':ti,ab OR 'hypoglycemia':ti,ab OR 'hypoglycaemia':ti,ab OR 'nocturnal hypoglycemia':ti,ab OR 'nocturnal hypoglycaemia':ti,ab OR 'delayed hypoglycemia':ti,ab OR 'delayed hypoglycaemia':ti,ab OR 'glucose area under the curve':ti,ab OR 'glucose auc':ti,ab OR 'glycemic variability':ti,ab OR 'glycaemic variability':ti,ab OR 'continuous glucose monitoring':ti,ab OR 'cgm':ti,ab OR 'time in range':ti,ab OR 'time below range':ti,ab OR 'tir':ti,ab OR 'tbr':ti,ab)

# Supplementary Table S2. Study-level data extraction.

| **Study** | **Participants/baseline** | **Treatment/design** | **Exposure/comparator** | **Meal/timing** | **Hypoglycemia ascertainment** | **Evidence role** |
| --- | --- | --- | --- | --- | --- | --- |
| Rasmussen 1999 | n=12/11; middle-aged; BMI ~29; sex 7M/4F in fasting phase; HbA1c ~8.0% | Diet/oral agents; randomized crossover | 0.4 g/kg ethanol ± exercise; rest/exercise controls | Light meal, 48%E carbohydrate; 4 h | Acute nadir only; no overnight threshold | Special-context indirect |
| Bantle 2008 | n=18; non-insulin-treated; other baseline details NR | Random-order crossover | Wine 240 mL/24 g ethanol vs grape juice | Dinner to next morning | No nadir, time-below-range, or CGM threshold reported | Direct overnight |
| Dalgaard 2004 | n=11; 5F/6M; baseline age/BMI/HbA1c in source table | Diet/oral agents; insulin excluded; crossover | 40 g ethanol+50 g carbohydrate vs 120 g carbohydrate | High-fat mixed meal; 8 h | Not prespecified | Direct meal; composition mismatch |
| Gin 1999 | n=30; age 53±6 y; BMI 27±2 | Diet/oral agents; parallel subgroups with self-control | Red wine 200 mL, ethanol 16 g, or tannic acid vs water/control | 600 kcal lunch; 65 g carbohydrate; 5 h | Not defined | Direct postprandial |
| Christiansen 1994 | n=10; baseline details NR | Randomized crossover | 24 g ethanol vs control beer | Standardized light meal; 4 h | Not defined | Direct postprandial |
| Schaller 2010 | n=12 men; age 64±6 y; BMI 28.4±5.7; HbA1c ~7.4% | Diet/oral agents; open self-control | Vodka/40 g ethanol vs baseline | Fasting FSIGT | Not a clinical hypoglycemia study | Mechanistic indirect |
| Zhi 2016 | n=18; BMI eligibility 20–45; other baseline details in source | Drug-interaction crossover | 40 g ethanol+piragliatin vs drug/placebo conditions | With meals; 0–6 h | Not primary/prespecified for this review | Drug-context indirect |
| Avogaro 2004 | n=8; age ~58 y; BMI ~29; HbA1c ~8.2% | Crossover FSIGT | Vodka/40 g ethanol vs water | Fasting FSIGT | Not a clinical hypoglycemia study | Mechanistic indirect |
| Rasmussen 2001 | n=8; diet-treated; baseline details NR | Randomized crossover clamp | 0.4 g/kg ethanol vs water | Fasting clamp; 7 h | Experimental insulin-induced hypoglycemia | Mechanistic indirect |
| Foot 1997 | n=23; 17M/6F; age 43–74 y | Diet-treated; parallel drug+crossover challenge | 0.6 g/kg ethanol vs control under troglitazone/placebo | Dinner; 4 h and next morning | Symptoms and urinary cortisol:creatinine; no CGM threshold | Drug-context indirect |
| Gin 1992 | n=10 non-insulin-treated subgroup; men; other details NR | Random self-control | Red wine, 2 glasses vs same lunch | Lunch | Definition NR | Direct; limited report |
| Koivisto 1993 | n=16; age 57±2 y; BMI 27.2±1.0; mostly diet/oral treated | Randomized crossover | Ethanol 1 g/kg vs water/control meal | Dinner to morning; 45%E carbohydrate meal | No morning value <5 mmol/L; no overnight CGM definition | Direct next-morning |
| Christiansen 1993 | n=12; median age 55 y; BMI 26; mostly diet/oral treated | Four-period crossover | Dry/sweet wine vs water/glucose-matched conditions | Light meal; 4 h | Not defined | Direct postprandial |
| Chellingsworth 1990 | n=9; 8M/1F; age 58.6±3.9 y; BMI 28.2±0.7 kg/m²; HbA1c 7.8±0.6%; five diet, three metformin, one chlorpropamide | Randomized-order two-evening within-person crossover | Standard meal with 50 mL sherry and 260 mL wine; sweet vs dry beverages; no alcohol-free control | Sherry 0–30 min; meal/wine 30–90 min; snack 150 min; glucose to 240 min and prebreakfast ≈14 h | Serial samples to 240 min plus one prebreakfast sample; no continuous overnight monitoring; authors reported no hypoglycemia | Direct meal-associated active comparator; beverage composition only |
| Hosaka 2008 | n=6 men; diet-treated T2D; age 60±15 y; BMI 21.7±1.8 kg/m²; duration 10.6±5.2 y; HbA1c 6.4±0.4% | Three-condition within-person comparison on different days; sequence/randomization NR | Beer 800 mL, sake 300 mL, or shochu 180 mL; glucose contents 24.8, 14.7, and 0 g; no alcohol-free control | After 12–14 h fast; glucose at 0, 30, 60, 120 min | No definition/event ascertainment; no overnight or next-morning follow-up | Fasting indirect active comparator; beverage composition only |

# Supplementary Table S3. Selected glucose outcome data after unit harmonization.

| **Study** | **Outcome** | **Comparison** | **Result** | **Interpretation** |
| --- | --- | --- | --- | --- |
| Rasmussen 1999 | Glucose iAUC, 0–240 min | Ethanol+exercise vs rest | 509 ± 98 vs 724 ± 109 mmol/L·240 min | Lower vs rest; exercise confounded |
| Gin 1999 | Peak excursion, 90 min | Red wine vs water | 1.8 ± 0.9 vs 2.6 ± 0.8 mmol/L | Lower with red wine |
| Christiansen 1994 | Glucose iAUC, 0–240 min | Ethanol vs control | 574 ± 89 vs 554 ± 69 mmol/L·240 min | No significant difference |
| Koivisto 1993 | Next-morning fasting glucose | Alcohol vs water | Paired difference −0.7 ± 0.2 mmol/L | Lower; no morning hypoglycemia |
| Foot & Eastmond 1997 | Next-morning glucose | Alcohol vs control | 6.7 vs 6.8 mmol/L (troglitazone group) | No significant difference reported |
| Zhi 2016 | Glucose AUC ratio, 0–6 h | Ethanol+piragliatin vs piragliatin | 1.13 vs 1.00 | 13% increase; drug context |
| Chellingsworth 1990 | Glucose AUC 0–240 min; prebreakfast ≈14 h | Sweet vs dry wine and sherry | AUC 31.4±2.37 vs 29.01±1.32 mmol/L·h (mean±SE; p=0.25); prebreakfast 7.70±0.77 vs 7.36±0.51 mmol/L; no participant reported hypoglycemic | No significant AUC difference; prebreakfast paired inference NR; no alcohol-free control |
| Hosaka 2008 | Serial glucose 0–120 min | Beer vs sake vs shochu | Beer 0/30/60/120 min: 5.78±0.16/9.17±0.66/10.83±0.88/9.23±0.36 mmol/L; sake: 5.77±0.24/7.24±0.98/8.40±0.50/7.22±0.44; shochu: 5.78±0.18/5.94±0.63/5.89±0.29/5.81±0.35 | Beer and sake rose from baseline; shochu did not; no between-beverage test or alcohol-free control |

# Supplementary Table S4. Risk-of-bias judgments.

RoB 2 domain-level judgments. Ratings: Low risk, Some concerns, or High risk. Figure 3 presents the corresponding traffic-light plot.

| **Study** | **Randomization** | **Period/carryover** | **Deviations** | **Missing data** | **Outcome measurement** | **Selective reporting** | **Overall** |
| --- | --- | --- | --- | --- | --- | --- | --- |
| Rasmussen 1999 | Some concerns | Some concerns | Some concerns | Low | Low | Some concerns | Some concerns |
| Bantle 2008 | Some concerns | Some concerns | Low | Low | Low | Some concerns | Some concerns |
| Dalgaard 2004 | Some concerns | Low | Some concerns | Low | Low | Some concerns | Some concerns |
| Christiansen 1994 | Some concerns | Some concerns | Low | Low | Low | Some concerns | Some concerns |
| Avogaro 2004 | Some concerns | Low | Low | Low | Low | Some concerns | Some concerns |
| Rasmussen 2001 | Some concerns | Low | Low | Low | Low | Some concerns | Some concerns |
| Foot 1997 | Some concerns | Some concerns | Some concerns | Some concerns | Low | Some concerns | Some concerns |
| Gin 1992 | Some concerns | Some concerns | Some concerns | Some concerns | Low | High | High |
| Koivisto 1993 | Some concerns | Low | Low | Low | Low | Some concerns | Some concerns |
| Christiansen 1993 | Some concerns | Some concerns | Low | Low | Low | Some concerns | Some concerns |
| Chellingsworth 1990 | Some concerns | Some concerns | Low | Low | Low | Some concerns | Some concerns |

**Supplementary Table S4b. ROBINS-I domain-level judgments.**

| **Study** | **Confounding** | **Selection** | **Classification** | **Deviations** | **Missing data** | **Outcome measurement** | **Selective reporting** | **Overall** |
| --- | --- | --- | --- | --- | --- | --- | --- | --- |
| Gin 1999 | Moderate | Moderate | Low | Low | Low | Low | Moderate | Moderate |
| Schaller 2010 | Serious | Low | Low | Moderate | Low | Low | Moderate | Serious |
| Zhi 2016 | Moderate | Moderate | Low | Moderate | Low | Low | Moderate | Moderate |
| Hosaka 2008 | Serious | Moderate | Low | Moderate | Low | Low | Moderate | Serious |

# Supplementary Table S5. Certainty of evidence using GRADE considerations.

Certainty was assessed qualitatively using the GRADE domains of risk of bias, inconsistency, indirectness, imprecision, and publication bias. No pooled effect estimate was calculated. The ratings indicate confidence in the direction and magnitude of the evidence, not an estimate of clinical benefit or harm.

| **Outcome/question** | **Evidence base** | **Reasons for downgrading** | **Certainty** | **Interpretation** |
| --- | --- | --- | --- | --- |
| Postprandial glucose response | 8 direct meal studies (one active-beverage comparison) plus one fasting composition study | Risk-of-bias concerns; serious inconsistency and imprecision; active comparators and fasting context add indirectness | Very low | No consistent improvement; new data reinforce beverage carbohydrate/matrix as modifiers, not an ethanol class effect. |
| Overnight glucose stability or nocturnal hypoglycemia | Four studies extending into the evening or next morning; no adequate T2D-specific overnight CGM analysis | Very serious indirectness and imprecision; Chellingsworth had no measurements from 4 to 14 h; event ascertainment limited | Very low | Evidence neither demonstrates nor reliably excludes nocturnal hypoglycemia. |
| Next-morning fasting glucose | One small alcohol-versus-water crossover plus one sweet-versus-dry active-comparator crossover and other non-replicating comparisons | Very serious imprecision and inconsistency; no overnight CGM; active comparison cannot estimate alcohol effect; lack of replication | Very low | The −0.7±0.2 mmol/L finding remains confined to one 1993 alcohol-versus-water study; the active comparison neither replicates nor refutes it. |
| Mechanistic counterregulation and special contexts | Clamp/FSIGT, exercise, drug-interaction, and fasting beverage studies | Indirect to meal-associated overnight use; small samples; co-interventions and active comparators | Very low | Alcohol may affect substrate handling, insulin sensitivity, and glucagon response; beverage carbohydrate affects early glucose, but clinical overnight events are not established. |

# Supplementary Table S6. Full-text exclusions and unavailable reports.

| **Report** | **DOI/identifier** | **Decision** | **Reason** |
| --- | --- | --- | --- |
| Effects of Red Wine and Daily Exercise on Glycemic Control in Insulin Resistant Individuals | 10.70252/ijes2026203 | Excluded | Mixed T2D/prediabetes results reported together; T2D effect not separable |
| Acute effects of graded alcohol intake on glucose, insulin and free fatty acid levels in non-insulin-dependent diabetic subjects | No DOI identified | Not retrieved | Full text could not be obtained. Corresponding author contacted by email; no response received by 31 August 2026. |
| Alcohol tolerance in patients with non-insulin-dependent (type 2) diabetes treated with sulphonylurea derivatives | PMID 8053971; Embase L24210105; no DOI | Not retrieved | Re-identified in Embase; full text unavailable; companion PMID 8415209 not counted separately. Corresponding author contacted by email; no response received by 31 August 2026. |
| Alcohol Drinks Induce Acute Lowering in Circulating L-Arginine in Obese and Type 2 Diabetic Subjects | 10.1089/jmf.2021.0045 | Not retrieved | Full text could not be obtained. Corresponding author contacted by email; no response received by 31 August 2026. |
| 'Diabetes mellitus and alcohol. Relationship between hypoglycemia and lipid metabolism' | Embase L13033267; no DOI | Not retrieved | Full text unavailable after Embase and web searching; abstract-only results were not synthesized. Corresponding author contacted by email; no response received by 31 August 2026. |
| The Effect of Ethanol on Overnight Glucose Regulation in Type 2 Diabetes Mellitus | NCT00566592 | Excluded: registry only | No posted results or linked result-bearing publication; duplicate Embase register record not counted again |
| Wine Consumption and Glycemic Control | NCT02518334 | Excluded: registry only | No posted results or linked result-bearing publication |

**Supplementary Table S7. Structured effect-direction and heterogeneity matrix.**

| **Study** | **Evidence stratum** | **Exposure** | **Comparator** | **Outcome/time** | **Available statistics** | **Effect direction** | **Synthesis implication** |
| --- | --- | --- | --- | --- | --- | --- | --- |
| Rasmussen 1999 | Special context | 0.4 g/kg ethanol+exercise | Rest/exercise | iAUC, 0–240 min | Means±SE; paired variance NR | Lower vs rest; no clear difference vs exercise | Exercise confounding; not poolable |
| Bantle 2008 | Direct overnight | Wine 24 g ethanol | Grape juice | Evening-to-morning curves | Detailed paired variance NR | No clear difference | Different beverage/comparator and outcome format |
| Dalgaard 2004 | Direct meal | 40 g ethanol+50 g carbohydrate | 120 g carbohydrate | Glucose, 8 h | Time-series; paired variance NR | No clear benefit | Meal composition mismatch |
| Gin 1999 | Direct postprandial | Red wine or isolated ethanol | Water/control | Peak excursion, 90 min | Means±SD; subgroup comparison | Lower with red wine; no clear difference with ethanol | Beverage-specific |
| Christiansen 1994 | Direct postprandial | 24 g ethanol | Control beer | iAUC, 0–240 min | Means±SE; paired difference SD NR | No clear difference | No compatible paired variance |
| Gin 1992 | Direct postprandial | Red wine | Same meal | Glucose curve | Numerical data incomplete | Favored wine by author report | High risk/selective reporting |
| Koivisto 1993 | Direct next morning | 1 g/kg ethanol | Water/control meal | Fasting next morning | Paired difference±SE | Lower | Only direct positive morning study |
| Christiansen 1993 | Direct postprandial | Dry/sweet wine | Water/glucose-matched | Median iAUC, 4 h | Medians/IQR | No clear overall difference | Incompatible summary metric |
| Foot 1997 | Drug context | 0.6 g/kg ethanol | Control under drug/placebo | 4 h and next morning | Means; dispersion NR | No clear difference in drug group | Drug context; not poolable |
| Zhi 2016 | Drug context | 40 g ethanol+piragliatin | Piragliatin | AUC, 0–6 h | Ratio; CI NR | Higher | Pharmacodynamic interaction |
| Schaller 2010 | Mechanistic | Vodka/40 g ethanol | Baseline/control | Fasting FSIGT | Means±SE | Higher insulin sensitivity | Indirect outcome |
| Avogaro 2004 | Mechanistic | Vodka/40 g ethanol | Water | Fasting FSIGT | Means±SE | Higher insulin sensitivity | Indirect outcome |
| Rasmussen 2001 | Mechanistic | 0.4 g/kg ethanol | Water | Clamp recovery/glucagon | Means±SE | Lower glucagon; similar recovery | Indirect outcome |
| Chellingsworth 1990 | Direct meal/next-morning active comparator | Sweet wine and sherry | Dry wine and sherry | Glucose AUC 0–240 min; prebreakfast ≈14 h; hypoglycemia | Means±SE; paired-difference variance NR | No clear difference | Addresses beverage composition, not alcohol vs no alcohol; not poolable |
| Hosaka 2008 | Fasting indirect active comparator | Beer, sake, or shochu | Other alcohol-containing beverages; no non-alcoholic control | Serial glucose 0–120 min | Means±unclear dispersion; within-baseline p values; no pairwise condition test | Higher after beer and sake; stable after shochu | Response tracked carbohydrate; fasting context and serious bias prevent causal inference or pooling |
